# Eye-Strain as a Cause of Disease

**Published:** 1915-11-06

**Authors:** 


### EYE-STRAIN AS A CAUSE OF DISEASE.

### FACTORS IN SUCCESSFUL TREATMENT.

The term "eye-strain" may be defined as the condition which exists when abnormal efforts have to be made to bring rays of light reflected from external objects to a focus on the retina. In a normal (emmetropic) eye rays of light from a distance of, say, more than six metres are focussed without any effort; and for near objects the effort demanded is well within the power of the healthy accommodative mechanism. This latter statement is at least true until about forty-five years of age, but after this age the mechanism has failed to such a degree that the eye cannot easily focus the divergent rays which it receives from near objects (presbyopia), and the patient therefore needs the help of a convex glass, as in reading. Obviously, therefore, if he continues to dispense with this assistance he is likely to suffer from eye-strain. Again, in the hypermetropic eye, where the axis of the globe from before backwards is too short, there is a risk that rays of light entering the eyeball will be focussed not upon the retina but behind it, and to correct this result the patient will insensibly use his accommodative power. So long as the defect is not excessive and the patient is young no appreciable strain will be experienced. But as age advances and the power of accommodation fails strain will inevitably appear, just as it appears even in the normal eye, but now at an earlier date. Again, therefore, there is an opportunity for the development of eye-strain. Once more, suppose that the curved surfaces of the eyeball by which rays of light are bent to a focus have not all one and the same degree of curvature (astigmatism), and it is obvious that a sharp focus or clear image will not be formed on the retina, and that in the effort to secure this the patient is likely to suffer from eye-strain. Here then are three conditions—presbyopia, hypermetropia, and astigmatism—in each one of which there is undue effort made in the act of vision, and especially in the act of near vision, and in each of which, therefore, the consequences of this effort are more or less inevitable.

### Symptoms of Eye-Strain.

Now it is quite beyond doubt, and indeed it is in the very nature of things, that eye-strain arising in one or other of the fashions above described may cause local disturbances in the eyeballs themselves. These disturbances may take the shape of definite pain, or aching, or lacrymation, or blinking of the lids, or of recurrent inflammatory attacks. A point about the local pain is the frequency with which it is experienced in the early morning, and this time incidence often leads the patient to conclude that it cannot be his vision which is at fault. The pain, as he puts it, comes on after he has given his eyes a night's rest. It does not occur to him that muscular action—and this is the essential part of accommodative effort—can be accomplished without discomfort, at least for a time, and that the "stiffness" which results from it only becomes troublesome when, after a period of rest, the muscles are again called upon for renewed activity. What is true of an energetic game of football or cricket is true of an energetic use of the muscles of accommodation; the effects are experienced less at the moment than some hours later. Hence the fact that pain is not felt while the eyes are being used, as for reading, for example, does not by any means exclude eye-strain as its cause. Equally it must be said that the patient's protest that his vision is quite good is no proof that he is not suffering from the consequences of eye-strain. In the first place he may be mistaken, for having had all his days only one and the same pair of eyes he has not the materials for a comparative judgment. And it is the very essence of the case that he has been making undue efforts to secure good vision and that in this he may have succeeded; but his success is the fruit of strain and carries an appropriate penalty. No observer, therefore, should allow his suspicions of eye-strain to be lulled to rest either by an announcement that the vision is good or by a reference of the pain or discomfort to some period of the day when no special visual effort is in operation.

### Pitfalls to Avoid.

From pain or other discomforts in the eyeballs as a result of eye-strain it is no great step to the conclusion that in a similar manner may arise headaches of various degrees of severity. Undue efforts, especially when sustained or frequently repeated, may tell upon the brain as well as on the muscles, and therefore there is no startling quality in the statement that headache, migraine, or neuralgia may be the results of eye-strain. What has been said about local discomfort in the eyeballs may be repeated here. Neither the absence of any apparent association between the headache and the use of the eyes, nor the patient's conviction that his vision is perfect, forms a sufficient ground on which to dismiss eye-strain as the cause of his suffering. There is something more to add. Already the suggestion has been made that by the exercise of undue accommodative effort vision for practical purpose may be made to appear perfect even although one or other of the ocular defects mentioned in the first paragraph of this article undoubtedly exists. The defect is, as it were, overcome or concealed by the very strain which gives rise to the symptoms. Hence arises the necessity to determine the ocular facts under conditions which make this concealment impossible. In other words, the accommodative mechanism must often be paralysed as by atropine, homatropine, etc., in order that refractive error may be discovered. This doctrine is particularly true of children and young adults, in whom the power of accommodation is still strong, and though the middle-aged presbyopic may often escape the method, even he may at times have to yield to it. Again and again has the truth been proclaimed that neither the patient's sense of good vision nor his successful performance with standard test-types is inconsistent with the existence of eye-strain, and yet it is repeatedly forgotten. The only sure ground of exclusion is the examination of the eyes when atropine or some other cycloplegic has rendered the accommodative mechanism inactive.

Up to the point here stated there is hardly room for dispute or controversy. But the position is very different when the question is asked whether eye-strain may produce other effects than disturbances in the eyeballs and headaches. Here we are brought into contact with very large claims. According to some writers there is hardly any suffering or defect—mental, moral, or physical—which may not be caused by eye-strain and remedied by the use of appropriate glasses. From bronchial catarrh and pyelitis to insanity and suicide are included a programme of evils which may be explained and neutralised in this invitingly simple fashion. A well-known writer on the other side of the Atlantic has even analysed the lives of various well-known public men with a view to show that their peculiarities, their defects, their miseries, perhaps even their genius, were all alike the consequences of conditions which might well have been met by the art of the spectacle-maker. At first sight, no doubt, all this seems to carry a note of exaggeration or of extravagance, and on this ground alone the whole case is by not a few promptly dismissed as absurd. Now, it may be allowed that the man who is called an apostle by his followers, and by his critics a "crank," is usually inclined to over-statement, but the admission does not mean that his teaching has not some element of truth in it. And in medicine we are less concerned with large general propositions than with particular and individual instances. The question in practice is not, Can eye-strain cause this, that, and the other disturbance, but rather, Is a given patient suffering from eye-strain, and will the treatment of this relieve his sufferings? And the answer is not in doctrines or propositions, but in experience and in fact. Whether the general argument is true or false may engage academic discussion, but for the patient and his medical adviser the issue is both narrower and more easily resolved.

### The Personal Factor.

Among those who minimise the pathological possibilities of eye-strain it is customary to hear two statements which on the surface are not without plausibility. First it is said that as almost every eye has some degree of astigmatism it is absurd to suggest that mild degrees of this defect can in any individual carry important consequences; and a second comment is that not infrequently persons are found with a relatively high degree of astigmatism and yet entirely free from any of the symptoms of which eye-strain is accused to be the author. Neither of these objections, however, can be said to be valid. The first assumes that all individuals are substantially alike in their reactions to disturbing influences—a proposition which flies in the face both of medical knowledge and of common experience. It may be very unreasonable for Brown to lose his appetite and his sleep under the influence of anxiety, while Robinson, who is equally tried, continues to enjoy his five-course dinner and his eight hours' slumber—this may be unreasonable, but none the less it happens, and the only explanation available is that Brown is himself, and not Robinson. Equally it may be expected that a degree of astigmatism which to one man is a bagatelle may be to another an irritant almost beyond endurance; and the expectation is certainly justified by experience. On the objection that as high degrees of astigmatism sometimes exist apart from discomfort low degrees cannot possibly be of importance, the statement that has just been made may be repeated. But in addition it must be remembered that in the former the victim may never have known really clear vision, and that therefore he makes no strain to obtain it, while a slight astigmatism is easily neutralised in early life, and the individual having thus enjoyed good vision continues his effort to maintain this even when in the process of time his accommodative mechanism begins to decline.

### Reflex Influences.

There are two other general considerations which bear upon the disturbances for which eye-strain may be responsible. The first is that the possible play of reflex irritation acting through the nervous system may be very widespread, and neither physiologists nor physicians can with confidence put a limit to the extent of this influence. Add to this the fact that the use of the eyes during the greater part of the twenty-four hours scarcely knows rest, particularly in these days of much reading, and it is not difficult to imagine that eye-strain even of very moderate degree may have consequences both multiple and far-reaching. A second general comment is that the function of vision is not a detached and isolated performance, but is, on the contrary, related to and influenced by the state and working of other parts of the bodily machinery. Hence a degree of eye-strain which, existing as an individual defect may cause no symptoms, may yet become extremely troublesome when additional strains have to be borne. The astigmatism alone may be successfully met by the natural elasticity of a healthy nervous system, but all the same it may be a factor contributing to collapse when overwork or worry or dyspepsia also comes on the scene. If only the last straw had not been added the back of the camel would have sustained, and perhaps enjoyed, the previous burdens; and presumably if the latest contribution could be removed all would be well again. In plain language, it is necessary in the practice of medicine to take note both of the idiosyncrasies of the individual and of all, not merely of one, of the agencies which are working to his prejudice. This general doctrine applies emphatically to eye-strain. Because in individual patients this becomes operative only when associated with other evils it is none the less true that the treatment of it may break a vicious circle and may be the necessary influence for the relief of the patient.

